# Analysis of co-infection in severe and critical patients with influenza A (H1N1) pneumonia using metagenomic next-generation sequencing on bronchoalveolar lavage samples

**DOI:** 10.3389/fcimb.2025.1669328

**Published:** 2025-09-30

**Authors:** Weitao Gong, Xiaolin Ma, Gaoming Wang, Yongzhong Guo, Zhiyuan Zhuo, Conghui Han, Yanmin Wu

**Affiliations:** ^1^ Department of Pulmonary and Critical Care Medicine, Xuzhou Central Hospital, Southeast University Affiliated Xuzhou Central Hospital, Xuzhou Clinical School of Xuzhou Medical University, Xuzhou, Jiangsu, China; ^2^ Department of Neurology, Xuzhou Central Hospital, Xuzhou Clinical School of Xuzhou Medical University, Southeast University Affiliated Xuzhou Central Hospital, Xuzhou, Jiangsu, China; ^3^ Department of Thoracic Surgery, Xuzhou Central Hospital, Xuzhou Clinical School of Xuzhou Medical University, Southeast University Affiliated Xuzhou Central Hospital, Jiangsu, China; ^4^ Department of Urology, Xuzhou Central Hospital, Southeast University Affiliated Xuzhou Central Hospital, Xuzhou Clinical School of Xuzhou Medical University, Jiangsu, China

**Keywords:** influenza A (H1N1), co-infection, bronchoalveolar lavage fluid, metagenomicnext-generation sequencing, bronchoalveolar lavage

## Abstract

**Objectives:**

The study aimed to clarify the co-infection patterns in adult patients with severe influenza A (H1N1) pneumonia using Metagenomic Next-Generation Sequencing (mNGS) and to examine their impact on clinical outcomes, particularly focusing on the differences between severe and critical patient groups.

**Methods:**

This retrospective analysis evaluated bronchoalveolar lavage fluid (BALF) from 53 adult patients diagnosed with severe influenza A (H1N1) pneumonia. Patients were categorized into severe and critical groups depending on the need for invasive ventilation. mNGS was utilized to detect and analyze co-infections, which included fungal, bacterial and viral pathogens. Statistical analysis was conducted to assess the prevalence of these co-infections and their association with clinical outcomes, such as 28-day mortality.

**Results:**

In the cohort, 48 patients (90.6%) experienced co-infections. In the severe group, fungal infections were noted in 14 patients (66.7%), bacterial in 4 patients (19.0%), and viral in 11 patients (52.4%). Among the critical group, 22 patients (68.8%) had fungal, 23 patients (71.9%) had bacterial, and 10 patients (31.3%) had viral co-infections. There was a significantly higher incidence of co-infections in critical patients (*P* = 0.0002), with notable differences in *Acinetobacter baumannii* prevalence between the groups (*P* = 0.0339). *Aspergillus* emerged as the predominant fungal genus across the study. Septic shock (odds ratio [OR] 33.63[4.29−538.3]; *P* = 0.003) and fungal co-infection (OR 24.42[1.98−810.6]; *P* = 0.029) were identified as independent risk factors for 28-day mortality.

**Conclusion:**

The study revealed a high rate of co-infections in both severe and critical patients suffering from influenza A (H1N1) pneumonia, with a higher frequency of bacterial infections in critical patients. Importantly, septic shock and fungal co-infections were independently associated with increased 28-day mortality, highlighting the need for monitoring and management of co-infections in these patients.

## 1 Introduction

The influenza virus is an significant global health threat because it affects morbidity and mortality (Kumari et al., 2023), as exemplified by the 2009 appearance of the highly virulent H1N1 strain (Napolitano et al., 2014). In the 2009 pandemic, there were more than 18% of the patients who developed bacterial co-infections in combination with viral pneumonia (Martín-Loeches et al., 2011; Nin et al., 2011). Evidence suggests that bacterial co-infections, rather than the virus itself, are an important cause of influenza-induced death (Brundage and Shanks, 2008; Chien et al., 2009). Aspergillus species were also identified as significant co-infection pathogens (Martin-Loeches and Valles, 2012; van de Veerdonk et al., 2017). Nonetheless, the rate of co-infection and the associated risk factors remain poorly delineated.

The pandemic underscored the need for efficient influenza surveillance systems and accelerated the pace of pathogen detection technology development. In this regard, Metagenomic Next-Generation Sequencing (mNGS) stands out, integrating high-throughput sequencing with sophisticated bioinformatics analysis (Gu et al., 2021). Several advantages make mNGS attractive, such as quick detection, sequencing DNA or RNA from small amounts of clinical samples, and the capacity for parallel detection of many different pathogens (Chen et al., 2021). All these advantages favor the use of the technology for the detection of rare, new, and unknown causes, as well as for the detection of complicated infectious conditions with uncommon origins. As a newer technology, however, mNGS is costly and has unknown clinical correlations. Moreover, its application in analyzing bronchoalveolar lavage fluid (BALF) from patients with influenza A (H1N1) pneumonia complicated by respiratory failure has not been thoroughly studied in the literature.

This analysis used mNGS to determine the actual rate and variation of co-infection in the BALF microbiome among patients classified according to differing disease severity levels in influenza A (H1N1) pneumonia, notably those who were severely and critically ill. In addition, the analysis sought to determine the specific risk determinants for co-infection and investigate the association with outcomes in mortality.

## 2 Materials and methods

### 2.1 Research subjects and data gathering

This observational study, conducted retrospectively, concentrated on adult patients aged 18 and older, who were admitted to the hospital with a confirmed diagnosis of Influenza A (H1N1) Pneumonia. The study was conducted following the guidelines outlined in the “Chinese Expert Consensus on the Imaging Diagnosis of Severe Pneumonia Resulting from Influenza A (H1N1)”. Data were collected from patients admitted to Xuzhou Central Hospital, a 4,500-bed university-affiliated medical center, during the period from October 2024 to March 2025. The data collected included demographic details, infection symptoms, the interval from symptom onset to hospital admission, laboratory test outcomes, complications, and death status within a 28-day period. To assess disease severity, researchers computed the CURB-65 score (age ≥65, respiratory rate, urea, blood pressure, confusion) for all patients.

Inclusion criteria for this study encompassed patients diagnosed with Influenza A (H1N1) Pneumonia who met any of the following conditions: (1) oxygen saturation below 90% at rest, (2) the ratio of arterial partial pressure of oxygen (PaO2) to the fraction of inspired oxygen (FiO2) below 300 mmHg, or (3) progressive deterioration of clinical symptoms, demonstrated by imaging that reveals more than a 50% progression of lung lesions within 24 to 48 hours.

Patients were excluded if they met any of the following conditions: (1) were younger than 18 years old or were admitted from nursing homes or other healthcare facilities; (2) had bronchoalveolar lavage fluid collected more than 12 hours post-intubation;, or (3) had incomplete clinical information.

Patients were categorized based on clinical criteria into either a severe group or a critical group. The critical group included those experiencing respiratory failure, necessitating invasive mechanical ventilation.

The study protocol was approval from the Ethical Committee of Xuzhou Central Hospital, and all procedures adhered to the Declaration of Helsinki. To ensure confidentiality, patient identities were anonymized throughout the study. Consent was obtained from the guardians or family members of all participating patients.

### 2.2 BALF collection

Local anesthesia of the respiratory tract was achieved using 1% lidocaine, and an appropriate amount of midazolam administered intravenously. Patients were positioned in a supine orientation, after which alveolar lavage was conducted utilizing an electronic bronchoscope. A total volume of 100 mL of sterile normal saline was introduced, with each aliquot comprising 20 mL. Upon completion and recovery, preliminary quality control of specimens was carried out. In order to qualify, specimens were required to satisfy the following criteria: (1) absence of atmospheric secretions in the lavage fluid; (2) a recovery rate exceeding 40% and viability of living cells over 95%; (3) fewer than 10% red blood cells and less than 5% epithelial cells; (4) intact and undeformed cell morphology combined with uniform distribution. Samples that met these criteria were subsequently stored at −80 °C before being dispatched for laboratory analysis.

### 2.3 mNGS

The mNGS was conducted following the method previously described (Fang et al., 2021; Wu et al., 2024). The main steps are as follows: (1) Extraction of nucleic acids: The total DNA of the BALF sample was extracted using the QIAamp DNA Micro Kit (Tiangen, Beijing, China) in accordance with the reagent instructions. (2) Library preparation and sequencing were carried out. For library construction, 10 ng of DNA was utilized with the NEB Next^®^ Ultra™ DNA Library Prep Kit for Illumina^®^. The quality of the library was evaluated using the Agilent 2100 bioanalyzer. The quality of the library was evaluated using the Agilent 2100 bioanalyzer (Agilent Technologies). The library concentration was evaluated using the Qubit 2.0 (Thermo Fisher). Qualified libraries were pooled and loaded onto a Illumina Next-seq platform for sequencing. (3) Bioinformatics analysis: The raw sequencing data were initially processed using bcl2fastq2 to demultiplex and convert the data into a usable format. Subsequent quality control was performed with Trimmomatic to eliminate low-quality reads, remove adapter sequences, duplicates, and reads shorter than 36 base pairs. To exclude human reference genome sequence, the reads were aligned to the human reference genome (hg38 utilizing Bowtie2, and only those reads that did not map to the human reference genome were remained for further analysis. These unmapped reads were then compared with microbial databases available at ftp://ftp.ncbi.nlm.nih.gov/genomes/ using the Burrows-Wheeler Aligner (BWA). Taxonomic classification of the microbial reads was conducted with Kraken 2.0, and the resulting taxonomic report was refined using the Bracken Bayesian algorithm to achieve the relative abundance estimates of the species.

### 2.4 Definitions

Co-infection was considered suspected if a patient exhibited an acute onset of signs and symptoms indicative of a lower respiratory tract infection, along with radiographic evidence of a pulmonary infiltrate without any other known cause (Mandell et al., 2007). To confirm co-infection, laboratory confirmation following the Centers for Disease Control and Prevention criteria was required. Results from mNGS were regarded as “significant clues” rather than definitive “judgments.” The diagnosis of co-infection involving bacteria, fungi, and atypical pathogens follows the previously described criteria (Martin-Loeches and Valles, 2012; Martin-Loeches et al., 2017). For DNA viruses, particularly herpesviruses, a co-infection was considered “probable” if the bronchoalveolar lavage fluid (BALF) viral load was ≥ 10,000. For RNA viruses such as influenza, respiratory syncytial virus (RSV), parainfluenza virus, and adenovirus, a viral load of ≥ 100 indicated a potential co-infection. In this study, the determination of co-infection pathogens was based on a combination of clinical features (patient’s symptoms, signs, immune status, biomarkers like procalcitonin, and response to treatment), microbiological characteristics (pathogen virulence, sequence count, relative abundance, and microbial community structure), and “gold standard” criteria (positive smear, culture, or pathological findings). This comprehensive decision-making process relied on discussions among clinicians, microbiologists, infectious disease specialists, and laboratory personnel, integrating all available information.

### 2.5 Statistical analysis

Statistical analysis was performed using GraphPad Prism, version 10.1.2. For categorical variables, either the Fisher’s exact test or Chi-squared test was used. Continuous variables were summarized using the interquartile range (IQR) and the median and compared with the Mann-Whitney U test. To identify variables predictive of patients’ 28-day mortality, multivariate logistic regression analyses were conducted for all patients. The independent variables analyzed included the duration from onset to admission, smoking, blood lymphocyte count, septic shock, viral co-infection, bacterial co-infection, and fungal co-infection. A *P*-value of less than 0.05 was considered statistically significant.

## 3 Results

### 3.1 General characteristics

In this study, 53 patients with severe influenza A (H1N1) pneumonia were enrolled. Patients were stratified based on their clinical needs: 32 patients required invasive mechanical ventilation necessitating ICU admission, and were thus classified as the critical group. The remaining 21 patients were classified as the severe group.

The primary demographic and clinical traits of the study populations are outlined in **Table 1**. No notable statistical differences were observed between the severe and critical groups in terms of gender, age, severe obesity (BMI ≥ 40), smoking history, types of comorbidities, and the duration from onset to admission. Furthermore, the main clinical symptoms, such as cough, fever, and asthma, did not show significant differences between the two groups. In contrast, a statistically significant difference was noted between the severe and critical groups in the incidence of septic shock and 28-day mortality.

**Table 1:** Demographic and clinical traits.

Characteristics	Influenza A (H1N1) pneumonia patients			*P*-value
	Total (N = 53)	Severe group (N = 21)	Critical group (N = 32)	
Male, n (%)	33 (62.3)	13 (61.9)	20 (62.5)	0.965
Age (years), median (IQR)	67.8 (63–76)	67.6 (62-70)	68 (62-77)	0.616
Severe obesity (BMI ≥ 40), (n, %)	3 (5.7)	1 (4.8)	2 (6.3)	0.999
Smoking, n (%)	32 (60.4)	12 (57.1)	20 (62.5)	0.696
Hypertension, n (%)	10 (18.9)	6 (28.6)	4 (12.5)	0.169
Diabetes, n (%)	13 (24.5)	5 (23.8)	8 (25)	0.922
Chronic bronchitis, n (%)	23 (43.4)	10 (47.6)	13 (40.6)	0.615
Cerebral infarction, n (%)	9 (17.0)	3 (14.3)	6 (18.8)	0.999
Cough, n (%)	53 (100)	21 (100)	32 (100)	0.999
Fever, n (%)	42 (79.2)	18 (85.7)	24 (75)	0.494
Asthma, n (%)	53 (100)	21 (100)	32 (100)	0.999
From onset to admission (days), median (IQR)	6.6 (4-8)	6.8 (5-9)	5.8 (4-7)	0.246
Septic shock, n (%)	15 (28.3)	0	15 (46.9)	0.0001
Died in 28d, n (%)	7 (26.4)	0	14 (43.8)	0.0006

IQR, interquartile range; BMI, Body Mass Index; *P*-value, between the severe and critical groups. Statistical significance between the severe and critical groups was determined by Fisher’s exact test or Chi-squared test for categorical variables, or by Mann-Whitney U test for continuous variables.

As shown in **Table 2**, no significant differences were observed in axillary temperature, white blood cell count, or IL-6 levels between the severe and critical groups. In contrast to the severe group, the critical group demonstrated statistically significant changes characterized by a lower blood lymphocyte count, decreased HS-CRP levels, and elevated PCT levels. Furthermore, the GM positivity rate was higher in the critical group compared to the severe group, with the serum GM positivity rate being notably more pronounced. The difference in serum GM positivity reached statistical significance.

**Table 2:** Initial clinical assessment upon admission.

Examination	Influenza A (H1N1) pneumonia patients			*P*-value
	Total (N = 53)	Severe group (N = 21)	Critical group (N = 32)	
Axillary temperature (°C), median (IQR)	38.0 (37.8-38.7)	38.1 (37.9-38.6)	38.0 (36.7-38.9)	0.839
White blood cell count, median (IQR)	9.6 (8.0-12.3)	9.7 (8.6-12.3)	9.5 (6.9-12.3)	0.4621
Blood lymphocyte count, median (IQR)	0.73 (0.46-0.90)	0.81 (0.49-0.95)	0.69 (0.35-0.71)	0.033
HS-CRP (mg/L), median (IQR)	89.3 (44.8-142.5)	110.1 (58.7-160.7)	75.7 (41.1-124.7)	0.018
PCT (mg/L), median (IQR)	1.13 (0.06-0.53)	0.13 (0.05-0.16)	1.78 (0.07-0.71)	0.003
IL-6 (ng/L), median (IQR)	97.2 (16.3-166.1)	110.0 (15.7-203.0)	75.7 (15.6-144.3)	0.932
Serum GM, n (%)	25 (47.2)	3 (14.3)	22 (68.8)	0.0002
BAL GM, n (%)	27 (50.9)	7 (33.3)	20 (62.5)	0.0514
CURB-65 score, median (IQR)	3 (2-4.5)	2.14 (2-2)	3.56 (3-4.5)	<0.0001

IQR, interquartile range; HS-CRP, high-sensitivity C-reactive protein; PCT, procalcitonin; IL-6, interleukin- 6; CURB-65, age ≥65, respiratory rate, urea, blood pressure, confusion; GM, Galactomannan; *P*-value, between the severe and critical groups. Statistical significance between the severe and critical groups was determined by Fisher’s exact test or Chi-squared test for categorical variables, or by Mann-Whitney U test for continuous variables.

### 3.2 BALF microbiota profiling

**Figure 1:**
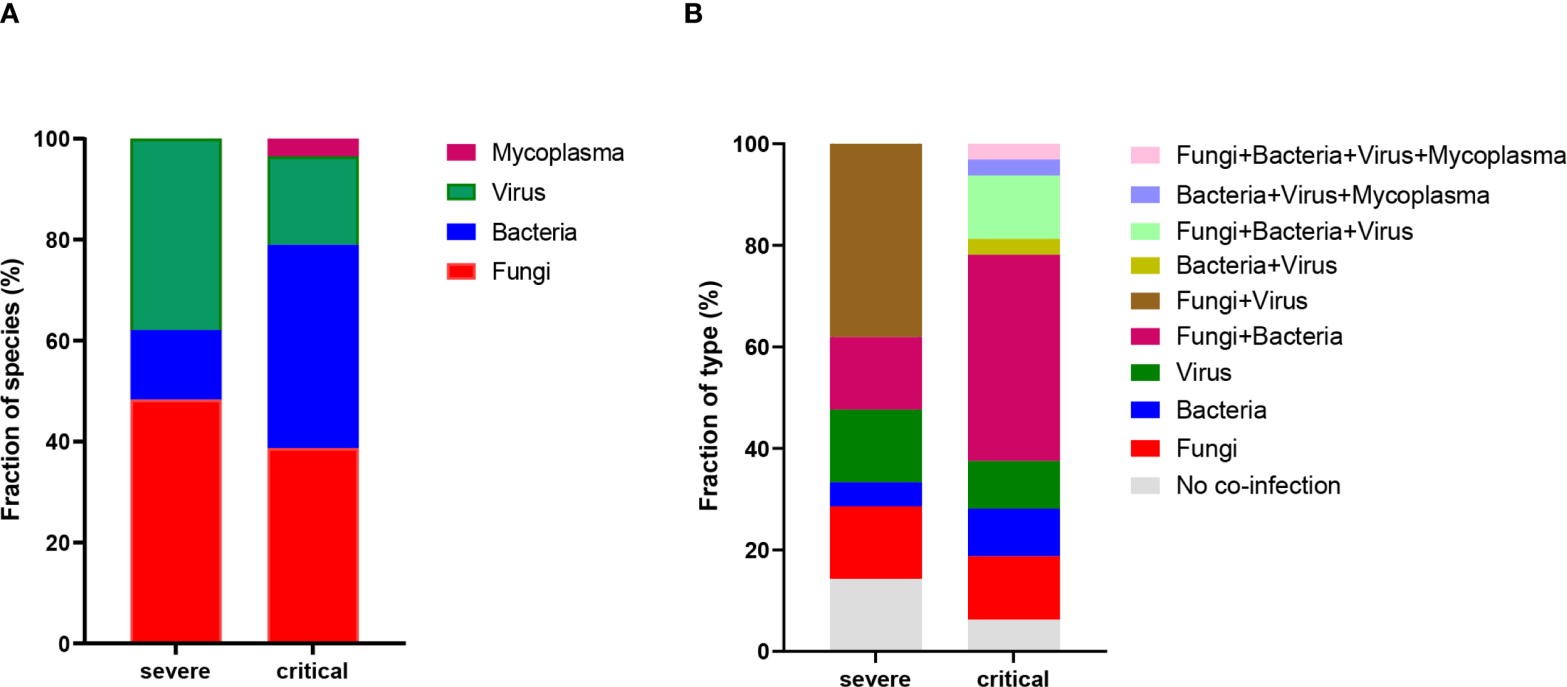
Profiling of BALF microbiota. **(A)** Statistics on the fraction of species present in patients from the severe and critical groups. **(B)** Percentage distribution of infection types in patients from the severe and critical groups.

This study, which involved 53 patients with influenza A (H1N1) pneumonia, observed differences in co-infection patterns between the severe and critical groups. In the severe group, only 3 patients (14.3%) were infected exclusively with the influenza A (H1N1) virus, while in the critical group, only 2 patients (6.3%) had sole infections. mNGS results showed that, in the severe group, 14 patients (66.7%) had co-infections involving fungi, 4 patients (19.0%) had bacterial co-infections, and 11 patients (52.4%) were co-infected with viruses. In contrast, in the critical group, 22 patients (68.8%) had fungal co-infections, 23 patients (71.9%) had bacterial co-infections, and 10 patients (31.3%) had viral co-infections, as illustrated in **Table 3**. Importantly, the rate of bacterial co-infection was significantly greater in the critical group compared to the severe group (*P* = 0.0002). However, no statistically significant differences were found between the two groups concerning fungal, viral, and mycoplasma co-infection rates. Specifically, in the severe group, the proportions of co-infections with fungi, bacteria, and viruses were 48.3%, 13.8%, and 37.9%, respectively. In the critical group, these proportions were 38.7%, 40.3%, 17.5%, and 3.5% for fungi, bacteria, viruses, and mycoplasma, respectively, as depicted in **Figure 1A**. As illustrated in **Figure 1B**, significant differences existed in the types of infections between the groups: the severe group predominantly experienced co-infections with fungi and viruses, whereas the critical group was primarily affected by co-infections with fungi and bacteria.

**Table 3:** Infections rate of co-infection pathogens.

Coinfection pathogens	Influenza A (H1N1) pneumonia patients			*P*-value
	Total (N = 53)	Severe group (N = 21)	Critical group (N = 32)	
No co-infection, n (%)	5 (9.4)	3 (14.3)	2 (6.3)	0.3739
Bacteria, n (%)	27 (50.9)	4 (19.0)	23 (71.9)	0.0002
Fungi, n (%)	36 (67.9)	14 (66.7)	22 (68.9)	0.8737
Viruses, n (%)	21 (39.6)	11 (52.4)	10 (31.3)	0.1240
Mycoplasmas, n (%)	2 (3.8)	0 (0)	2 (6.3)	0.5123

Statistical significance between the severe and critical groups was determined by Fisher’s exact test or Chi-squared test.

**Figure 2:**
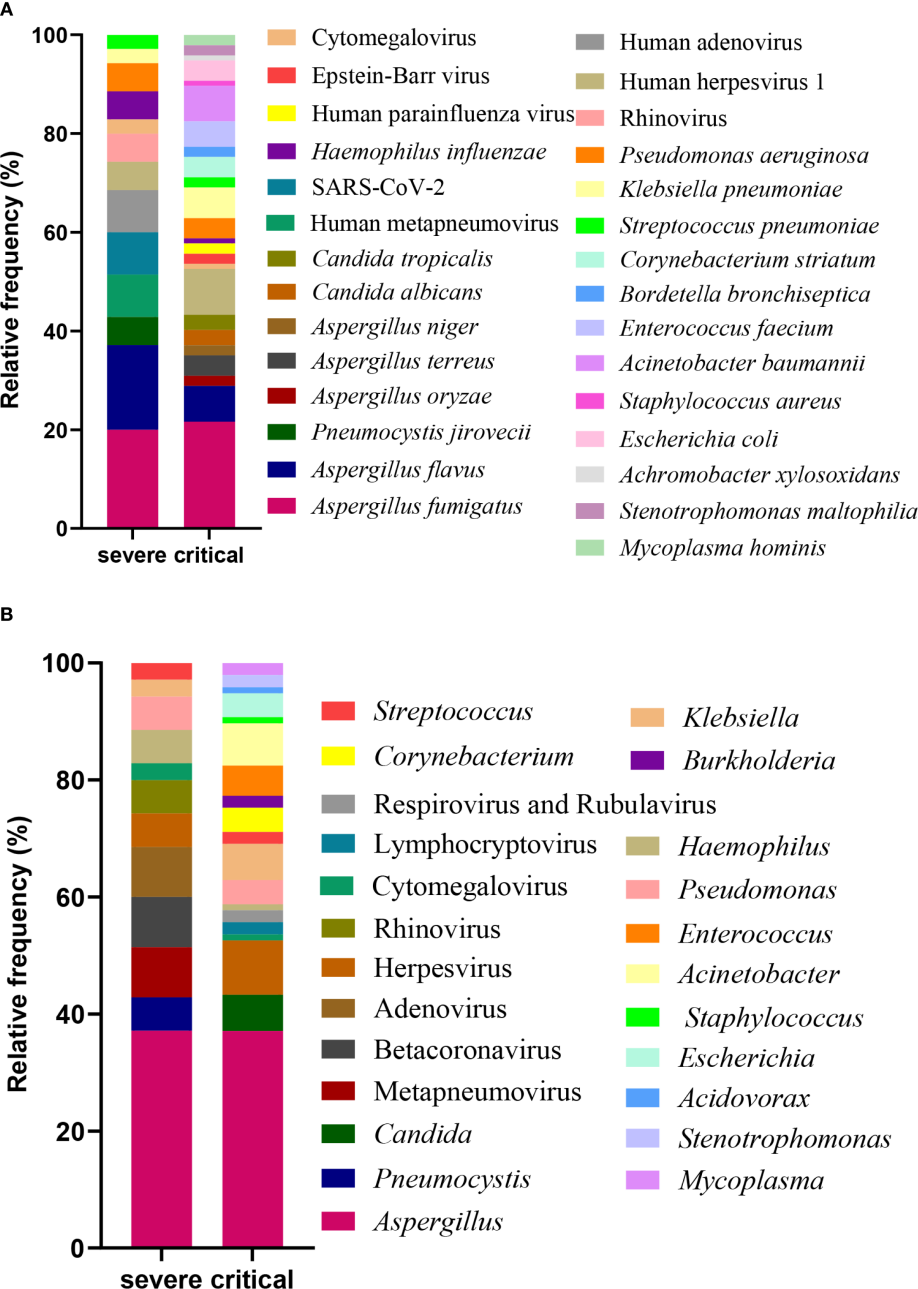
Profiling of BALF microbiota. The relative frequency of species **(A)** and genus **(B)** observed in patients from the severe and critical groups.

Following the exclusion of potential background microorganisms (as detailed in **Supplementary Table S1**, **Supplementary Table S2**), **Figure 2A** presents an overview of the predominant bacteria, viruses, fungi, and mycoplasmas identified in BALF at the species level. Within the severe group, notable species included *Aspergillus fumigatus*, *Aspergillus flavus*, Human metapneumovirus, SARS-CoV-2, Human adenovirus, *Pneumocystis jirovecii*, Human herpesvirus 1, Rhinovirus, *Pseudomonas aeruginosa*, *Haemophilus influenzae*, *Klebsiella pneumoniae*, *Streptococcus pneumoniae*, and Cytomegalovirus. Among these, *Aspergillus fumigatus* was the most prevalent fungal species, while *Haemophilus influenzae* and *Pseudomonas aeruginosa* were the leading bacterial species. In terms of viral species, Human metapneumovirus, SARS-CoV-2, and Human adenovirus were predominant. In contrast, the critical group exhibited a different spectrum of major species, which included *Aspergillus fumigatus*, Human herpesvirus 1, *Aspergillus flavus*, *Klebsiella pneumoniae*, *Acinetobacter baumannii*, *Enterococcus faecium*, *Escherichia coli*, *Pseudomonas aeruginosa*, *Aspergillus terreus*, *Corynebacterium striatum*, *Candida albicans*, *Candida tropicalis*, *Aspergillus oryzae*, *Aspergillus niger*, Epstein-Barr virus, Human parainfluenza virus, *Stenotrophomonas maltophilia*, *Mycoplasma hominis*, *Streptococcus pneumoniae*, *Bordetella bronchiseptica*, *Haemophilus influenzae*, *Staphylococcus aureus*, Cytomegalovirus, and *Achromobacter xylosoxidans*. In this cohort, *Aspergillus fumigatus* was identified as the dominant fungal species, *Acinetobacter baumannii* as the principal bacterial species, and Human herpesvirus 1 as the leading viral species.

**Figure 2B** presents the predominant genera of bacteria, viruses, fungi, and mycoplasmas identified in BALF specimens. In patients in the severe group, the ten most frequently detected genera were *Aspergillus*, Metapneumovirus, Betacoronavirus, Adenovirus, *Pneumocystis*, Herpesvirus, Rhinovirus, *Haemophilus*, *Pseudomonas*, and *Klebsiella*. Comparatively, in the critical group, the top 10 genera comprised *Aspergillus*, Herpesvirus, *Acinetobacter*, *Candida*, *Klebsiella*, *Enterococcus*, *Escherichia*, *Corynebacterium*, *Pseudomonas*, and *Stenotrophomonas*. *Aspergillus* was consistently identified as the dominant fungal genus across both patient groups. *Haemophilus* and *Pseudomonas* emerged as the predominant bacterial genera in the severe group, while *Acinetobacter* was the principal bacterial genus in the critical group. Regarding viral genera, Metapneumovirus, Betacoronavirus, and Adenovirus were prevalent in the severe group, whereas Herpesvirus was the leading genus in the critical group.

### 3.3 Differences in taxa between the severe and critical groups

**Figure 3:**
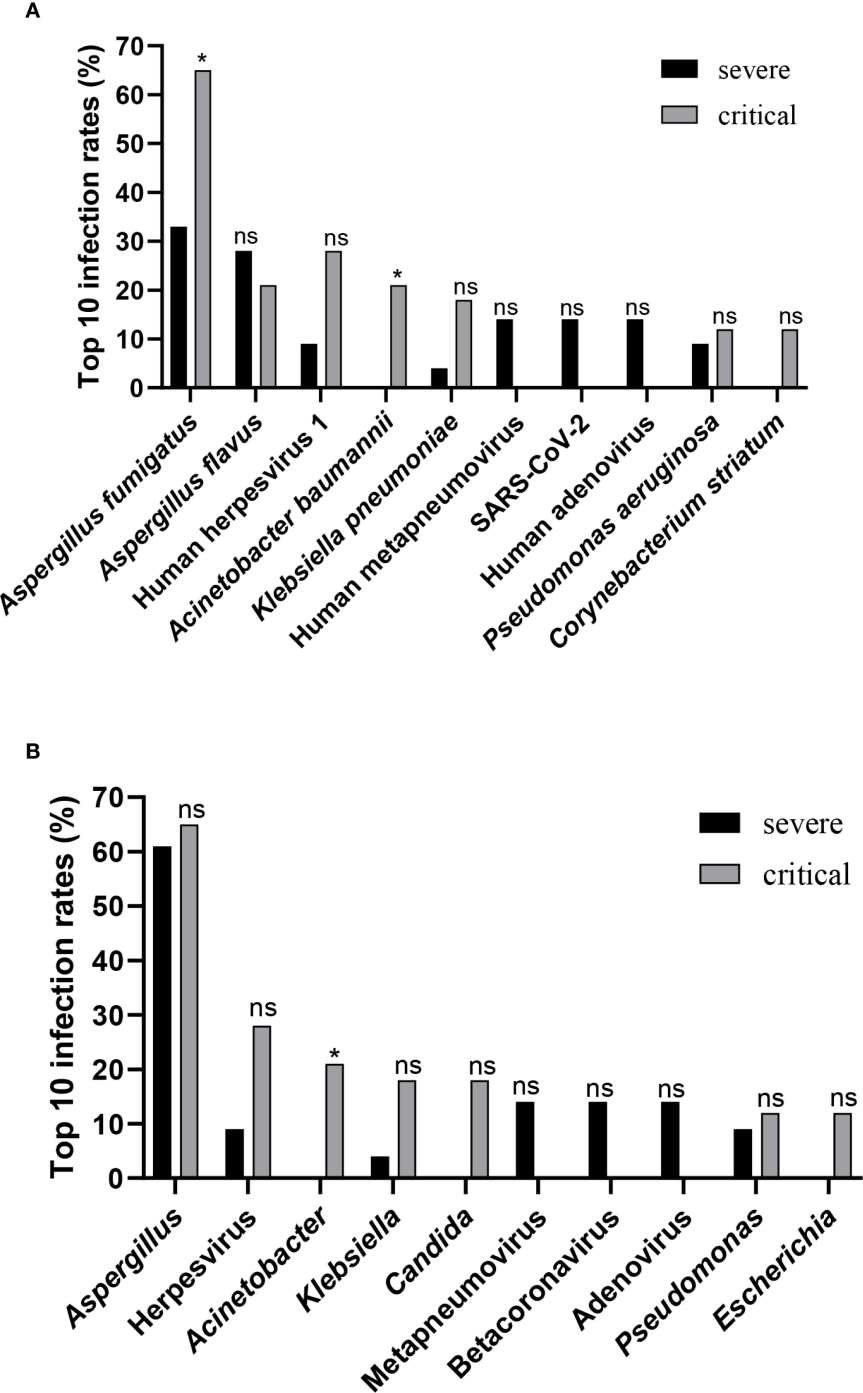
Differences in taxa between groups. **(A)** Comparison of infection rates between the severe group and the critical group, targeting the top 10 species-level taxa. **(B)** Comparison of infection rates between the severe group and the critical group, focusing on the top 10 genus-level taxa. Statistical significance in panels **(A, B)** was determined by Fisher’s exact test or Chi-squared test (ns, not significant; *P < 0.05).

The relative frequencies of the top 10 taxa were analyzed and compared between the severe and critical groups. Human metapneumovirus, SARS-CoV-2, and Human adenovirus were more prevalent in the severe group than in the critical group; however, these differences did not reach statistical significance. Conversely, the relative frequency of *Aspergillus fumigatus*, Human herpesvirus 1, *Acinetobacter baumannii*, *Klebsiella pneumoniae*, and *Corynebacterium striatum* were significantly lower in the severe group compared to the critical group, with statistically significant differences observed for *Aspergillus fumigatus* and *Acinetobacter baumannii* (**Figure 3A**). Furthermore, whileapneumovirus, Beta coronavirus, and Adenovirus were more prevalent at the genus level in the severe group compared to the critical, these differences were also not statistically meaningful. In contrast, the genera Herpesvirus, *Acinetobacter*, *Klebsiella*, *Candida*, and *Escherichia* showed notably lower frequency in the severe group relative to critical group, with the difference *Acinetobacter* achieving statistical significance (*P* = 0.0339) (**Figure 3B**).

### 3.4 Clinical outcomes

**Figure 4:**
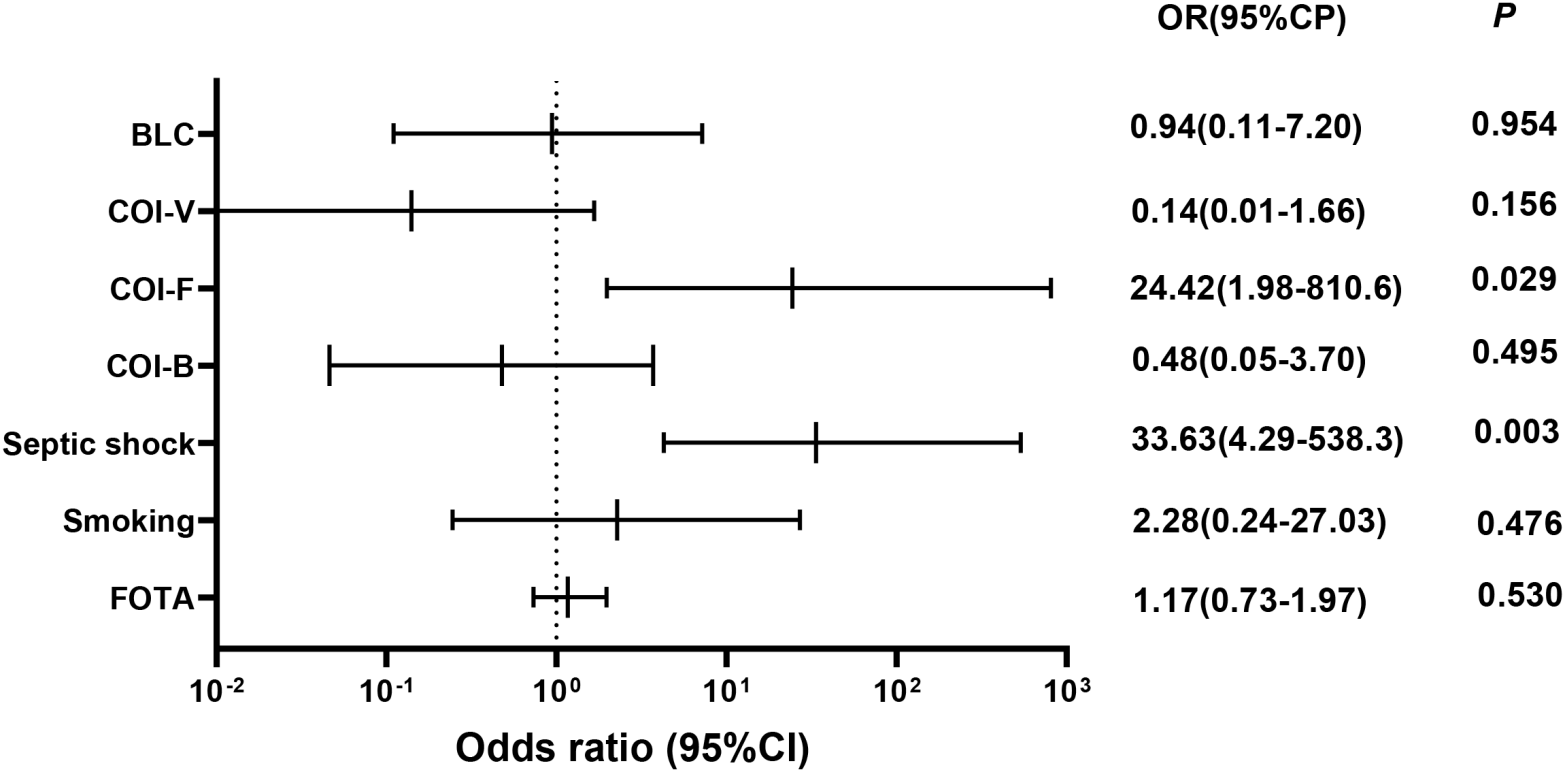
Odds ratios for risk factors associated with 28-day mortality. BCL, blood lymphocyte count; COI-V, viral co-infection; COI-F, fungal co-infection; COI-B, bacterial co-infection; FOTA, from onset to admission. Multivariate logistic regression analyses were conducted for all patients to identify variables that predict 28-day mortality.

The analysis indicated that there was no meaningful variation in 28-day mortality concerning factors such as the duration from onset to admission, smoking, blood lymphocyte count, viral co-infection, and bacterial co-infection (**Figure 4**). However, septic shock (OR 33.63[4.29−538.3]) and fungal co-infection (OR 24.42[1.98−810.6]) were found to be independent factors associated with increased 28-day mortality (*P* = 0.003; *P* = 0.029).

## 4 Discussion

Influenza viruses exemplify significant human pathogens due to their recurrent seasonal epidemics and the continuous threat of pandemics (Damak et al., 2011; Vicari et al., 2021). Annually, seasonal influenza-related illnesses result in over 650,000 global deaths (Iuliano et al., 2018). The mortality rate for hospitalized influenza patients stands at 4%, escalating to 20%-25% among severe ICU cases (Verweij et al., 2020). A primary cause of mortality in influenza patients is the development of co-infections after the initial viral illness, with bacterial and fungal co-infections being prevalent complications among critical patients (Quah et al., 2018; Qiao et al., 2023; Lu et al., 2024). Diagnosing and managing these co-infections is both complex and crucial, as timely intervention can substantially reduce mortality rates in those with complications post-influenza (Sarda et al., 2019). Consequently, successful treatment hinges on accurately identifying the pathogens responsible for co-infections in patients with differing severities of influenza pneumonia.

Bronchoalveolar lavage fluid serves as an optimal sample for the analysis of inflammatory and immune cells, cytological assessments, and microbial etiologies of pulmonary diseases at the alveolar level (Meyer, 2007). Its widespread use in diagnostic testing enhances the identification of pathogens involved in lung diseases, offering greater accuracy than samples like sputum or blood (Iroh Tam et al., 2015; Li et al., 2020). In this study, we used mNGS to examine the composition and diversity of the BALF microbiome in patients with influenza A (H1N1) pneumonia. The principal finding of study was the high rate of complicated co-infections, which substantially worsened the clinical course in the patients.

Bacterial co-infection in influenza patients has an important bearing on the clinical condition, with typical common pathogens including *Pseudomonas aeruginosa, Streptococcus pneumoniae, Haemophilus influenzae*, and methicillin-sensitive *Staphylococcus aureus (* Joseph et al., 2013; Teng et al., 2019; Bartley et al., 2022). This study determines the common bacterial pathogens after ruling out the possibility of background contaminants and categorizes them in relation to the disease intensity. In the severe group, the common infectious agents were *Pseudomonas aeruginosa, Haemophilus influenzae, Streptococcus pneumoniae*, and *Klebsiella pneumoniae*. Conversely, in the critical group, pathogens such as *Corynebacterium striatum*, *Achromobacter xylosoxidans*, *Enterococcus faecium*, *Acinetobacter baumannii*, *Klebsiella pneumoniae*, *Streptococcus pneumoniae*, *Staphylococcus aureus*, *Haemophilus influenzae*, *Escherichia coli*, *Pseudomonas aeruginosa*, and *Stenotrophomonas maltophilia* were prevalent. A meta-analysis and comprehensive review involving 27 papers exhibited wide variability in the rates of bacterial co-infection in influenza patients, ranging from 2% to 65%, whereas the vast majority reported 11% to 35% (Klein et al., 2016). There has been an increase in the rates in the case of influenza patients, based on historical data, from 11.4% in 2009 to 23.4% in 2015 (Martin-Loeches et al., 2017). Bacterial co-infection was identified in 19% of the cases in severe group in the present investigation and rose sharply to 71.9% in the cases in critical group, whereas *Acinetobacter baumannii* exhibited high variance in the two groups. Additionally, compared to the severe group, the critical group exhibited a significant increase in PCT levels and a higher incidence of septic shock. In this study, bacterial co-infections did not demonstrate an independent association with mortality rates. Nonetheless, in instances of severe bacterial co-infections, especially those involving septic shock, septic shock was independently linked to increased mortality. The results of this study concerning the association between bacterial co-infection and mortality and between septic shock and mortality in H1N1 influenza align with the findings reported by Antoni Torres et al (Cillóniz et al., 2012).

This study found that critical patients with influenza A (H1N1) pneumonia are more susceptible to bacterial co-infections compared to those in the severe group. This pattern of co-infection impacts the disease’s pathogenesis and patient prognosis. Influenza A (H1N1) already imposes a considerable challenge to the host’s immune system (Short et al., 2016). The presence of bacterial co-infections can further strain the immune response, potentially leading to an excessive inflammatory state or immune paralysis, thereby advancing disease progression (McCullers, 2006). This may result in more severe lung injuries and increase the likelihood of complications such as acute respiratory distress syndrome (ARDS) in critical patients (Morris et al., 2017). Moreover, Influenza A (H1N1) infections can disrupt normal microbial homeostasis in the lungs, facilitating pathogen colonization and infection (Hanada et al., 2018). Data also suggest that bacterial co-infection may contribute to the progression of the illness from severe to critical. Patients with severe influenza are prone to secondary bacterial pneumonia, which can lead to clinical deterioration and necessitate mechanical ventilation, thus categorizing them as critically ill (Viasus et al., 2011). Most importantly, bacterial infections heighten the risk of septic shock, which may accelerate the transition from severe to critical illness (Angus and van der Poll, 2013).

While there were notable differences in the rates of bacterial infections and septic shock between severe and critical patient groups, 28-day mortality was associated solely with septic shock and not with bacterial co-infections. This may be attributed to several factors: Firstly, although bacterial infections are prevalent in critical patients, not all infections are highly lethal (Singer et al., 2016). Some bacterial infections may be more easily treatable or responsive to common antibiotics, thus not directly increasing mortality. Secondly, the severity of bacterial infections, particularly severe co-infections, can lead directly to septic shock, linking them to higher mortality rates (Antinori et al., 2020). Additionally, comorbidities play a significant role in critical patients (Chow et al., 2019). These individuals often face multiple health challenges, such as immunosuppression, underlying diseases, or increased pathogen exposure. Therefore, increased mortality may result from the combined effects of these factors rather than bacterial infections alone. Furthermore, effective therapeutic interventions are crucial in reducing mortality related to bacterial infections (Evans et al., 2021). In critical patients, healthcare teams may implement more aggressive strategies to identify and treat bacterial infections swiftly, such as using broad-spectrum antibiotics, thereby effectively lowering the risk of infection-related mortality (Seymour et al., 2017). In conclusion, while bacterial infections are common in critical patients, their impact on mortality may be mitigated by other factors. Further research is necessary to fully understand the complex interplay among these variables.

Fungal co-infections in influenza patients also have a significant impact on clinical outcomes, with *Aspergillus* species frequently identified as common pathogens (Beumer et al., 2019). A prospective, observational, multi-center study of 2,901 ICU patients with PCR-confirmed influenza employed standard microbiological methods to identify co-infections (Martin-Loeches et al., 2017). This study identified *Aspergillus* as the fourth most common pathogen in cases of severe influenza co-infections and highlighted that co-infections serve as an independent risk factor for mortality in the ICU. A multi-center retrospective cohort study in 2018 reported that the incidence of invasive pulmonary aspergillosis among patients with severe influenza in the ICU was 19%, with influenza being recognized as an independent risk factor for invasive pulmonary aspergillosis development (Schauwvlieghe et al., 2018). In the current study, fungal co-infections were detected in 66.7% of patients with severe illness and 68.9% of those with critical illness, with no statistically significant difference observed between the two groups. Among patients in severe group, *Aspergillus fumigatus* and *Aspergillus flavus* were commonly identified pathogens, and the Galactomannan positivity rates of serum and bronchoalveolar lavage fluid were significantly lower than the detection rate of mNSG. Similar to the severe group, *Aspergillus fumigatus* and *Aspergillus flavus* were also prevalent in the critical group. However, unlike the severe group, the Galactomannan positivity rates of serum and bronchoalveolar lavage fluid were close to the detection rate of mNSG. Studies show that in patients with normal immune function, the sensitivity of serum GM is significantly lower than that of BAL GM (de Oliveira et al., 2023). However, in patients with severely compromised immune systems, the positivity rate of serum GM starts to closely match that of BAL GM (Donnelly et al., 2020). This suggests that the statistical differences in serum GM observed between the severe and critically ill groups in this study may be linked to the extensive use of steroids and the severely weakened immune systems in critically ill patients. Consistent with the aforementioned study, this research found that fungal co-infection had an odds ratio (OR) of 24.42 and was identified as an independent factor associated with increased 28-day mortality.

The study demonstrates a complex relationship between co-infections and clinical outcomes in patients with severe influenza A (H1N1) pneumonia. Interestingly, while the prevalence of fungal co-infections is similar across both severe and critical patient groups, these co-infections are linked to a higher 28-day mortality rate. This seeming contradiction could be attributed to several factors. In patients with weakened immune systems, particularly those who are critically ill, fungal pathogens like *Aspergillus* might exhibit increased virulence, worsening the patients’ conditions and leading to poorer clinical outcomes (Bongomin et al., 2017). Moreover, fungal infections are often more difficult to diagnose and treat promptly compared to bacterial infections. Delays in initiating appropriate antifungal treatments can cause rapid clinical decline, especially in critical patients, thus elevating mortality rates (Szymański et al., 2022). Certain fungal co-infections, such as invasive *aspergillosis*, can result in severe systemic complications, thereby significantly increasing the risk of death, particularly in critical patients (Latgé and Chamilos, 2019). Fungal co-infections may also intensify the severity of bacterial or viral infections, resulting in more complicated clinical courses (Peleg et al., 2010). This synergistic effect might not change the infection prevalence but can have a considerable impact on mortality rates. Furthermore, the study highlights septic shock as an independent predictor of mortality, mainly occurring in critical patients, where fungal co-infections might induce or worsen shock conditions, leading to increased mortality rates. Additional investigation is required to thoroughly comprehend the intricate interactions between these variables.

In the context of co-infections involving influenza and COVID-19, substantial attention has been given to bacterial and fungal co-infections, whereas viral co-infections tend to be under-recognized due to technical limitations (Singh et al., 2021; Contes and Liu, 2025). In a study of 48 COVID-19 patients screened for 24 respiratory pathogens using six multiplex PCR panels, viral co-infections were detected, with Influenza A H1N1 and human adenovirus being the most frequently identified viruses (Alosaimi et al., 2021). Viral co-infections were associated with increased ICU admissions and higher mortality rates compared to bacterial co-infections. Notably, Influenza A H1N1 was the only pathogen directly related to increased mortality. During the influenza season, approximately 15% of patients exhibited co-infections with two or more respiratory viruses (Navarro-Marí et al., 2012). The primary co-infecting viruses included human rhinovirus, human metapneumovirus, respiratory syncytial virus, parainfluenza viruses, and adenovirus. There was no substantial link identified between co-infections of pandemic influenza with other respiratory viruses and the outcomes for influenza patients. In the present study utilizing mNGS technology, viral co-infections in influenza patients were found to play a significant role in lower respiratory tract infections. Viral co-infections were identified in 52.4% of patients with severe illness and 31.3% of those with critical illness, with no statistically significant difference between these two groups. Among patients with severe illness, human metapneumovirus, SARS-CoV-2, and human adenovirus were commonly detected pathogens. In critical patients, human herpesvirus 1 was notably prevalent. Compared to the severe group, patients in the critical group experienced a more significant decline in lymphocytes, which may be primarily related to the primary infection of influenza A (H1N1). Moreover, multivariate analysis also indicated that viral co-infection was not identified as an independent factor associated with increased 28-day mortality. In line with findings by E Cordero et al., it was observed that viral co-infection did not significantly influence the clinical outcome (Cordero et al., 2012).

Utilizing mNGS on bronchoalveolar lavage fluid, we conducted a retrospective study to assess co-infections in severe and critical patients with influenza A (H1N1) pneumonia. Through the analysis of co-infection patterns, this study offers several clinical benefits: (1) Accurate Diagnosis and Personalized Treatment: mNGS technology rapidly identifies multiple pathogens in a single sample, including bacteria, fungi, and viruses. This enables clinicians to quickly obtain a comprehensive infection profile, facilitating the implementation of more targeted and individualized anti-infective treatment strategies. (2) Optimization of Anti-infective Treatment Strategies: The study indicates that bacterial infections are more prevalent among critical patients, with a significant incidence of fungal co-infections, particularly *Aspergillus* infections. This insight helps guide the selection of initial antibiotic treatments, ensuring more effective coverage of common pathogens and potentially improving treatment outcomes. (3) Risk Assessment and Prognostic Prediction: Recognizing fungal co-infection and septic shock as independent risk factors for 28-day mortality provides crucial prognostic information for clinicians. By monitoring and addressing these risk factors, clinicians can identify high-risk patient groups and implement early interventions to reduce mortality risk and improve survival rates.This study recognizes several limitations. First, the relatively small sample size poses a constraint, especially given that microbial studies are subject to significant interindividual variability, and environmental and climatic factors may influence the outcomes. Moreover, clinical variables, such the administration of antibiotics before bacterial sampling, have the potential to alter the diversity of the patient’s microbiota. To substantiate our findings and conclusions, a cohort focusing on influenza A (H1N1) pneumonia is warranted. Second, because of the study’s retrospective and observational design, establishing cause-and-effect relationships between risk factors and outcomes is not feasible. Unidentified confounding variables, such as those linked to healthcare-associated pneumonia and the timing of antibiotic administration may still be present. As a result, our analysis was restricted to identifying correlations rather than conducting a causal investigation. Lastly despite analyzing the differences between and non-severe cases, a degree of bias is unavoidable.

## 5 Conclusions

This study reveals a significant prevalence of co-infections in patients suffering from severe and critical influenza A (H1N1) pneumonia, utilizing mNGS for precise pathogen identification. Our findings indicate a higher frequency of bacterial co-infections in critical patients, with *Acinetobacter baumannii* prevalence differing notably between severity and critical groups. In addition, *Aspergillus* co-infections were frequent in both patient groups and were associated with higher 28-day fatality rates. The findings indicate the interconnectedness of the co-infections in influenza A (H1N1) pneumonia and the significant contribution of co-infections toward clinical outcomes, emphasizing the imperative need for early pathogen detection. In spite of the constraint in the size of the analyzed samples and the retrospective and observational nature of the investigation, the observations favor the incorporation of mNGS in clinical practice as an excellent diagnostic tool. The procedure holds the promise for increasing diagnostic accuracy and guiding therapy, bettering the care of patients with influenza pneumonia complicated by co-infections. Larger cohort-based investigations in the future would help affirm the findings and determine the exact role of the application of mNGS in managing acute complicated infectious diseases.

## Data Availability

The original contributions presented in the study are included in the article/**Supplementary Material**. Further inquiries can be directed to the corresponding authors.
