# Supplementary material for: Analysis of co-infection in severe and critical patients with influenza A (H1N1) pneumonia using metagenomic next-generation sequencing on bronchoalveolar lavage samples
Source: Front Cell Infect Microbiol. 2025 Sep 30;15:1669328. doi: 10.3389/fcimb.2025.1669328 (PMC12518326; doi:10.3389/fcimb.2025.1669328)
Supplement: Supplementary file 1 [file Table1.docx]

Table S1 the potential background microorganisms in the severe group

| Name | | |
| --- | --- | --- |
| Streptococcus cristatus | Staphylococcus cohnii | Staphylococcus haemolyticus |
| Veillonella parvula | Corynebacterium tuberculostearicum | Streptococcus sanguinis |
| Candida parapsilosis | Cutibacterium acnes | Streptococcus_mitis |
| Candida albicans | Treponemavincentii | Actinomyces naeslundi |
| Malassezia restricta | Cardiobacterium hominis | Streptococcus infantis |
| Moraxella osloensis |  |  |

Table S2 the potential background microorganisms in the critical group

| Name | | |
| --- | --- | --- |
| Pseudomonas alcaligenes | Streptococcus salivarius | Schaalia odontolytica |
| Streptococcus mitis | Porphyromonas gingivalis | Veillonella parvula |
| Talaromyces marneffei | Streptococcus constellatus | Candida albicans |
| Tannerella forsythia | Streptococcus milleri | Pauljensenia hongkongensis |
| Cutibacterium acnes | Streptococcus intermedius | Cardiobacterium hominis |
| Limosilactobacillus fermentum | Staphylococcus haemolyticus | Ligilactobacillus salivarius |
| Lacticaseibacillus paracasei | Corynebacterium striatum | Actinomyces naeslundi |
| Streptococcus cristatus | Streptococcus oralis | Actinomyces naeslundi |
| TTV-like mini virus | Finegoldia magna | Capnocytophaga gingivalis |
| Abiotrophia defectiva | Rothia mucilaginosa | Tannerella forsythia |
| Mycobacteroides abscessus | Actinomyces israelii | Tropheryma whipplei |
| Human betaherpesvirus 7 | Campylobacter | Streptococcus_anginosus |
| Parvimonas micra | Rhodococcus qingshengii | Moraxella osloensis |
| Sphingomonas paucimobilis | Staphylococcus capitis | Elizabethkingia anophelis |
| Peptostreptococcus stomatis |  |  |
